# Influences of public health emergency and social isolation on older adults’ wellbeing: evidence from a longitudinal study

**DOI:** 10.3389/fpubh.2024.1417610

**Published:** 2024-08-29

**Authors:** Yuzhou Wang, Dong Zhou, Chen Wang

**Affiliations:** ^1^School of Urban and Regional Science, Shanghai University of Finance and Economics, Shanghai, China; ^2^Department of Cultural Industries and Management, School of Media and Communication, Shanghai Jiao Tong University, Shanghai, China

**Keywords:** social isolation, public health emergency, difference in difference, life satisfaction, a longitudinal study

## Abstract

Previous research has identified social isolation as a significant detriment to the wellbeing of older adults. However, studies that consider endogenous issues are scarce. The present paper examines the impact of the recent exogenous shock, the COVID-19 pandemic on the wellbeing of the older adult population using a longitudinal dataset from China for the period 2016–2020. The results of this study indicate that the life satisfaction of Chinese older adults was negatively affected, e particularly in regions where social distancing measures were more strictly enforced. Declines in physical and mental health were found to be attributable to declines in life satisfaction. Those who experienced greater exposure to the pandemic were more likely to suffer from chronic disease, illness, and insomnia, and many found it challenging to complete tasks during the lockdown. Furthermore, heterogeneity estimation shows that these effects are stronger among the rural older adult, females, those without a spouse, and those with less education.

## 1 Introduction

The recent public health emergency, COVID-19 pandemic and its associated social isolation policies, has been dramatically influencing human lives in diverse ways. Lifestyles and health conditions have been changed directly by the disease and the associated stabilization policies, as populations have been forced into sedentary lifestyles that have increased the incidence of mental illness (1–3). Meanwhile, rising unemployment and falling household incomes (4–8) as well as social distancing mandates have altered consumer purchasing behavior and household consumption in general (7, 9, 10). Research on individual subjective wellbeing has also emerged and found lower life satisfaction in regions subject to strict, comprehensive lockdown policies (11–13). However, one crucial aspect has still received scant scientific attention during the pandemics and lockdowns: psychological well-being among the older adults.

In 2020, the global population aged 65 and above was estimated to be approximately 737 million. A greater degree of psychological wellbeing among the older adult not only reflects more comprehensive social welfare but also contributes to economic growth. However, the older population is particularly susceptible to adverse effects during public health emergencies. The average age of those who have died from COVID-19 is over 70 years old. In general, the older adult is more likely to die and suffer more serious health damage after being infected than younger individuals (14–16). Concurrently, the concomitant lockdown policies inherently augment social isolation, whereas social engagement is crucial for wellbeing of the older adult. By examining these issues, our paper firstly fills a gap in the literature by reporting evidence of the effects of the current pandemic and lockdowns on the older adult. It extends the existing literature on the effects of the pandemic and lockdowns, but innovatively focuses on the life satisfaction and health of older adults, and empirically estimates the effects with advanced research designs.

Second, this paper complements the large body of literature that studies well-being and health outcomes of health risks caused by the contagious disease on the older adult. Over the past 100 years, the world has experienced several epidemics, including the 1918 flu, severe acute respiratory syndrome (SARS), avian influenza (H5N1), swine flu (H1N1), meningitis, Ebola, and the 2019 novel coronavirus (COVID-19). Globally and historically, these infectious diseases have threatened public health, economic development, quality of life, and so on across the globe (6, 17–20). During the COVID-19 pandemic, Chinese government has implemented strict and large-scale social distancing policies because of the pandemic and to some extent, the situation was unexpected and the older adults were positioned into a significant scenario of social isolation.

Social isolation has been well-documented as a significant factor influencing wellbeing of older adults (21). However, current measures of social isolation, such as living arrangements or infrequent contact with network members, have not fully addressed endogenous concerns, for example bidirectional relationships. Exogenous measures of social isolation and advanced research designs are needed to address these concerns and better understand the determinants of older adults’ wellbeing. The COVID-19 pandemic and its associated social isolation policies (e.g., lockdowns) provide a unique opportunity for causal investigation. Thus, thirdly we are able to provide the first attempt to measure social isolation with less endogenous concerns and adopt difference-in-differences (DiD) strategies to identify causal relationships between social isolation and well-being of the older adult, thereby contributing the literature on gerontology.

The primary objective of this study is to investigate the impact of the pandemic on the well-being outcomes of Chinese older adults. In order to empirically ascertain the impacts of the pandemic on the wellbeing outcomes of Chinese older adults, we employed a nationally representative longitudinal survey dataset: the 2016, 2018, and 2020 waves of China Family Panel Studies (CFPS). It encompassed a range of wellbeing outcomes, including life satisfaction, chronic disease, sickness, sleep difficulties, the frequency of feeling difficult to accomplish tasks, and loneliness. As reported, the 2019 novel coronavirus emerged in late December 2019 in Wuhan City. By late February 2020, however, the pandemic had been brought under control in most Chinese provinces. The 2020 wave of CFPS was collected after the initial outbreak during a relatively stabilized period during the pandemic. In parallel, the cumulative cases of and deaths from coronavirus infections in 2020 released by provincial public health commissions, permit the measurement of the extent of regional exposure. Consequently, the micro-panel data merged with regional infection data can be utilized to capture the within-person before-and-after effects of health risk factors across regions with different intense of exposure, including social isolation and the pandemic. The DiD estimators indicate that the older adult residing in regions with more severe infection rates and more stringent lockdown measures have experienced significantly greater declines in well-being because of physical and mental health concerns. This adverse impact is more pronounced among rural older adult individuals, female older adult individuals, those without spouses, and those with lower educational attainment.

The remainder of the paper is structured as follows. Section 2 presents the theoretical background and reviews associated literature. In Section 3 we describe the data for the analysis, the identification strategies, and the empirical models. Section 4 presents estimation results. Section 5 concludes.

## 2 Research background

### 2.1 The COVID-19 in China during 2019–2020

On December 8, 2019, the first pneumonia case of unknown cause was observed in Wuhan, the capital city of Hubei province, China. The pneumonia was later identified as caused by a new coronavirus (severe acute respiratory syndrome coronavirus 2, or SARS-CoV-2) (22), later named Coronavirus Disease 2019 (COVID-19) by the World Health Organization (WHO). COVID-19 was first reported to the local government on December 27, 2019 and, by January 29, 2020, the virus had spread into all provinces of mainland China, having radiated from Hubei province. Following January 23, 2020, all provinces immediately launched highest-level regulatory responses. China was the first country to impose drastic measures, including lockdowns and facemask mandates.

The Chinese government also adopted a “zero-COVID” strategy, which is designed to eliminate transmission of the virus within the country and allow normal economic and social activity to resume as quickly as possible (23). During this first wave of the pandemic, many regions homogeneously implemented strict anti-contagion policies, including strict social distancing, human mobility restrictions, and quarantine-on-entry policies, especially for residents from high-risk areas. Based on the “National overall emergency response plan for public emergencies” that was published in 2006, China divides public health emergencies into particularly serious (level I), serious (level II), major (level III) and general (level IV) levels, according to the nature, degree of harm, and scope of those emergencies. The most severe level is Level I, and the least severe level is Level IV.^1^ The timing of each province’s response time for moving to one level or another is listed in Supplementary Table 1.

**Figure 1:**
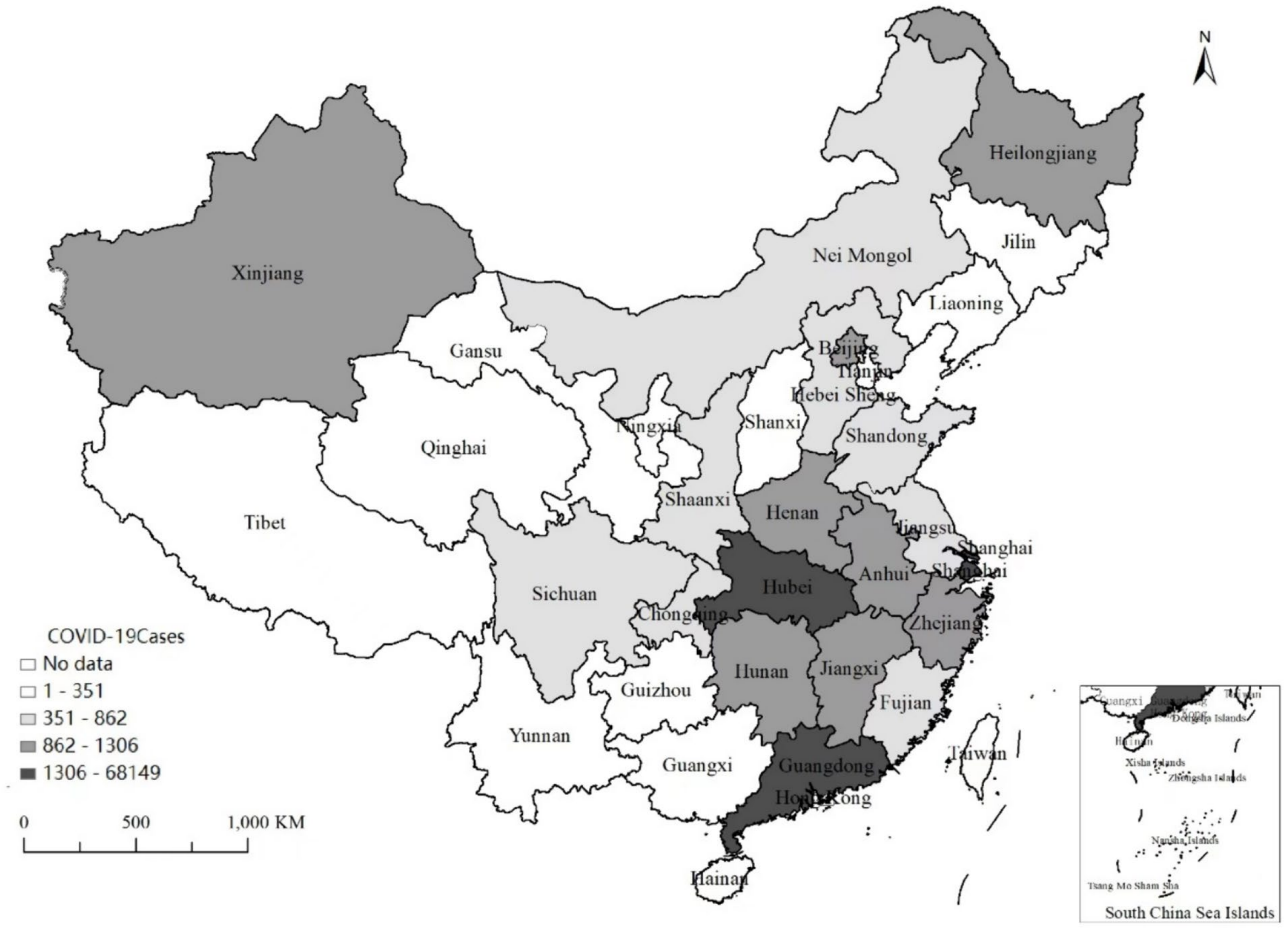
Geographical variation of the COVID-19 infection of mainland China 2019–2020. Most COVID-19 cases in Hubei, a central province of China, and significantly more cases in provinces adjacent to Hubei.

By late February, the pandemic had been brought under control in most Chinese provinces. On April 8, 2020, the lockdown was lifted in Wuhan where the coronavirus pandemic started. To some extent, this event represented the end of the first round of the COVID-19 outbreak in China, after which infections were scattered. After June 2020, all regulations were deescalated to the lowest level (level IV). Even though there were rising case numbers caused by international transmission sources in Heilongjiang and Xinjiang provinces, the national level was stable. As depicted in Figure 1 and Supplementary Table 2, reported COVID-19 cases varied across provinces in 2020 and so did the anti-contagion policies (24, 25). In late 2020, China’s economy continued its broad recovery from the recession that the pandemic triggered, with stable job creation and record international trade growth, although recovery in retail consumption remained slower than predicted.^2^

### 2.2 COVID-19 and wellbeing among older adults

Motivated by concerns for the wellbeing of the older adult, on May 12, 2022 we conducted a field study that involved surveying 1,207 Chinese individuals, including older adult individuals, in Beijing, Shanghai, and Jilin province, all of which were strongly affected by the Omicron spread in early 2022. We found that, during the lockdowns, 16.16% of surveyed residents experienced difficulties with medical treatment. Among those with common illness, 32.67% encountered difficulty seeking medical treatment, accounting for 5.2% of all residents interviewed. The older adult faced even greater difficulty, particularly with medical treatments and prescription medicines. Among the older adult, 68% reported psychological stress and even recurring neurological disease. Older adult individuals found it difficult to obtain staple goods. Unlike members of younger cohorts, older adults, especially those aged 65 years and above, are less digitally literate and depend heavily on governmental subsidies. Only 5.26% of the older adult population’s purchase goods online, while roughly one-quarter of other age cohorts do. The percentage of older people who lack necessities is much higher than in other groups.

Many studies have found a trade-off between widespread containment of infections and better wellbeing among the general population. For example, mobility restriction measures are found to be detrimental to psychological wellbeing and give rise to mental health problems (1, 3, 26). Schmidtke et al. (2) find reduced life satisfaction and mental health in 2020 after the first federal lockdown in Germany. Clark and Lepinteur (12) use longitudinal data collected from European countries during 2020 and find lower levels of life satisfaction in regions with stricter COVID-19 policies. Grimes (13) finds that individuals in New Zealand who live in stricter lockdown areas experience greater loneliness and lower life satisfaction than they did before the pandemic.

Clearly, in addition to the effects of infection *per se*, pandemics and lockdowns reduce social activities and physical exercise, and these reductions have been identified as important factors contributing to declining mental and physical health (11, 27, 28). For example, social activities have significantly positive impacts on cognitive function among the older adult (29). Lockdowns lead directly to reduced social interaction and greater loneliness. Reduced social interaction can deprive individuals of social resources and may reduce access to direct support for healthcare needs (30). Lockdowns can also predict more widespread feelings of isolation, which in turn predicts severer symptoms of depression and anxiety (31). Nevertheless, Kessler and Staudinger (32) suggest that, for the older adult, interacting with adolescents can compensate for age-related deficits (for example in cognitive performance and cognitive emotional complexity) and help increase the complexity involved in regulating emotions. Regarding loneliness, Hamermesh (33) points out that, when more time is spent alone, satisfaction diminishes. Loneliness affects neuroendocrine function and is associated with detrimental sleep patterns. Lonely individuals may also engage in worse health behaviors, such as poorer lifestyles, increased smoking and alcohol use, and less exercise, thereby possibly leading to cardiovascular disease and mental health difficulties (34). Health economists find that mental health problems rank among the top negative health conditions that undermine wellbeing (35–38). Consequently, it is postulated that the implementation of lockdowns will result in a reduction in life satisfaction among the older adult population, due to the deterioration of both physical and mental health.

## 3 Data, identification strategy, and model

**Figure 2:**
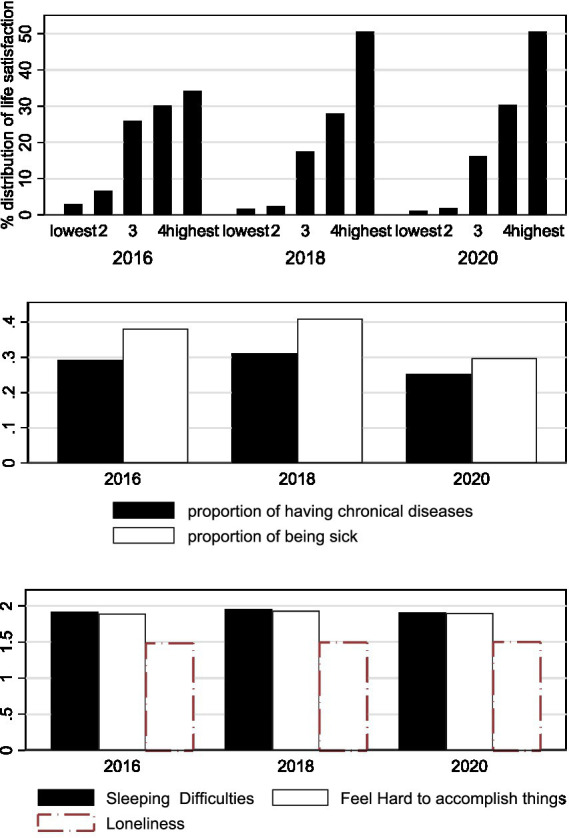
Distributions of life satisfaction and health outcomes 2019–2020. The average value of life satisfaction increased significantly from 2016 through 2018, and which increased greater for 2018–2020 period.

As mentioned above, we drew our empirical dataset from CFPS,^3^ and used the 2016, 2018, and 2020 waves with respondents above 60 years of age^4^. Applying personal identification numbers, we can track these individuals over time and construct a panel dataset for our empirical study. Information on coronavirus infection was collected from Tencent news and the data were released by the national as well as regional public health commissions. We merged the cumulative infected cases and deaths at the provincial level with the individual data. Our main dependent variable was life satisfaction measured as an ordinal variable on a 5-point scale ranging from the lowest to the highest degree of satisfaction. The mechanisms we examined included physical and mental health outcomes. The survey questions have asked respondents about the incidence of chronic disease during the previous half year, whether a respondent was ill during the previous 2 weeks, how often an individual encountered sleep difficulties per week, whether an individual had smoked within the previous 2 weeks, body mass index, the frequency with which it feels difficult to accomplish tasks, how often an individual has felt lonely, sad, and so on. In Table 1, we provided variable definitions and year-by-year descriptive statistics for all respondents aged 60 years and above. The overall statistics for the regression sample were presented in Supplementary Table 3 (the mean age is 68.13, ranging from 60 to 95; males accounts for 51% of the sample population and 48% are urban residents). The distribution of core variables over time was presented in Figure 2 and, in general, we did not observe significant declines or increases in the aggregated values in 2020. The average value of life satisfaction increased significantly from 2016 through 2018 (3.858 vs. 4.237), however, which was a much greater increase than that observed for the 2018–2020 period (4.237 vs. 4.277).

**Table 1:** Statistics description for all older adults at 60 and above.

All respondents ≥60	Year = 2016		Year = 2018		Year = 2020	
Variables	Mean	SD	Mean	SD	Mean	SD
Life satisfaction: 1–5 from the lowest to the highest						
	3.858	1.059	4.237	0.929	4.277	0.871
Social Status related to wealth and income: 1–5 from the lowest to the highest						
=1	0.120	0.325	0.068	0.252	0.055	0.228
=2	0.157	0.364	0.118	0.322	0.103	0.304
=3	0.406	0.491	0.380	0.485	0.371	0.483
=4	0.189	0.391	0.220	0.414	0.247	0.431
=5	0.129	0.335	0.215	0.411	0.224	0.417
Education attainment: 1–5 from the lowest to the highest						
*Below primary*	0.536	0.499	0.462	0.499	0.468	0.499
*Primary*	0.223	0.416	0.242	0.428	0.219	0.414
*Junior*	0.152	0.359	0.184	0.387	0.184	0.387
*Senior*	0.067	0.250	0.089	0.284	0.106	0.308
*Higher*	0.015	0.120	0.017	0.128	0.017	0.128
Health condition: 1–5 from the worst to the best						
=1	0.315	0.465	0.298	0.457	0.306	0.461
=2	0.232	0.422	0.170	0.376	0.164	0.371
=3	0.299	0.458	0.359	0.480	0.350	0.477
=4	0.095	0.293	0.091	0.288	0.092	0.290
=5	0.059	0.235	0.081	0.274	0.088	0.283
Marital status (1 Partner/Yes)	0.762	0.426	0.810	0.392	0.615	0.487
Severity	3.046	1.439	3.070	1.429	2.929	1.435
Age	69.333	7.529	68.224	6.338	70.105	7.367
Male	0.480	0.500	0.502	0.500	0.488	0.500
Urban Resident	0.472	0.499	0.484	0.500	0.480	0.500
Chronic disease: whether has got chronical disease during the past half year						
	0.265	0.441	0.311	0.463	0.222	0.416
Sickness: whether is sick or not in the last 2 weeks						
	0.352	0.477	0.425	0.494	0.267	0.443
Smoking: whether smoked during the past month						
	0.266	0.442	0.289	0.453	0.275	0.447
Feel hard to do everything: 1 = never; 2 = 1–2 days; 3 = 3–4 days; 4 = 5–7 days; frequencies in a week						
	1.895	0.992	1.933	0.992	1.902	1.015
Sleep difficulties: 1 = never; 2 = 1–2 days; 3 = 3–4 days; 4 = 5–7 days; frequencies per week						
	1.914	1.036	1.958	1.038	1.910	1.021
Loneliness: 1 = never; 2 = 1–2 days; 3 = 3–4 days; 4 = 5–7 days; frequencies in a week						
	1.484	0.820	1.495	0.832	1.500	0.847

Statistics description of regression sample in Table 2 are presented in Supplementary material.

The proportion for observations of having chronical diseases was about 0.3, and which of being sick was about 0.4. However, the latter was somehow low in 2020.

The frequency of facing difficulties to sleeping and to accomplishing things was about 2 out of 5, and which of feeling lonely was about 1.5 out of 5.

Our identification was essentially a DiD estimation; that is, we compared outcomes for individuals in regions that were more severely exposed to the COVID-19 pandemic and the associated regulations with outcome for those located in regions with less severe exposure. This identification strategy has been widely used with observational data, as in Duflo (39), Qian (40), and Lu and Yu (41). The 2020 CFPS wave was launched in the second half of 2020. Meanwhile, this was a period of temporary relief from COVID-19 and lockdown policies were loosened in most provinces. We measured outcomes in the 2020 wave after severe treatment and compared those outcomes with prior outcomes for each individual in the 2016 and 2018 waves. Using provincial numbers of infections and deaths in 2020, we then distinguished the varying severity of the spread of COVID-19 across regions and captured regional variations in associated anti-contagion measures. The timing of the survey and regional exposure severity made it possible to explore the causal effects of the pandemic on within-person wellbeing among the older adult through fixed-effects estimation.

Specifically, our specification for DiD estimation in the longitudinal study can be formulated as follows:


(1) $$\begin{array}{l} \mathrm{life}\;\mathrm{satisfactio}n_{iot}=\alpha +\lambda_{i}+\beta_{1}\mathrm{Interactio}n_{iot}\\ +\beta_{2}\mathrm{Exposure}\;\mathrm{Intensit}y_{\mathrm{io}}\\ +\beta_{3}\mathrm{coronavirus}\;year_{\mathrm{it}}\\ +\varphi X_{\mathrm{it}}+\emptyset_{t}+\delta_{o}+\psi_{j}+\varepsilon_{\mathrm{it}} \end{array}$$

where o indicates province o, i indicates individual i, j is the birth year cohort, and t represents survey year t. $\lambda_{i}$ represents individual fixed effects. Unobserved determinants of life satisfaction that are fixed at the provincial level, such as regional living standards, cultures, and customs, were controlled for through provincial indicators ( $\delta_{o}$ ). Similarly, members of our empirical sample population were born between 1921 and 1960 and have lived through major events including World War II, the founding of the modern Chinese nation, the Great Famine, and the Cultural Revolution. These event shocks can be absorbed by the birth cohort indicators ( $\psi_{j}$ ). *Exposure Intensity* measures the regional severity of exposure to the pandemic. Year indicators ( $\emptyset_{t}$ ) are also controlled for and we listed *coronavirus year* (which equals 1 if the survey time is 2020 and 0 otherwise) to measure the average within-person change during the pandemic. Interaction is measured by the product of *Exposure Intensity* and *coronavirus year*. X is a vector of control variables including age, gender, rural or urban residency, educational attainment, and social status related to personal wealth and marital status. $\varepsilon_{\mathrm{it}}$ is the error term. A significant coefficient of $\beta_{1}$ implies a significant effect of COVID-19 and anti-contagion policies on overall life satisfaction of the older adult, conditional on the overall covariates and fixed effects.

*Exposure Intensity* is constructed according to the severity of infections and measured by creating an ordinal variable comprising quantiles according to the regional population distribution of infections.^4^ Because infections in Hubei province exceeded the sum of all infections in the remaining regions, we set its value at 6. Also, we measured regional intensity directly as the natural log of infections or deaths, the results of which were shown in a robustness check. Moreover, we constructed alternative indicators representing whether a region was severely exposed to the pandemic (dummies were linked to regions with cumulative infections above 800 cases or numbers of deaths greater than 5, accounting for around 50% of the sample) and alternative ordinary exposure measures (say, creating 10 quantile categories). One concern was that our DiD estimates could be biased by unobserved major life events that occurred during this period other than COVID-19 that might drive changes in wellbeing, such as the death of a family member. Thus, we used a shorter panel that includes only the 2018 and 2020 waves to address these concerns. In addition, we analyzed subsamples with obvious differences in lockdown policies, compared regions distributed at the bottom 20% or 40% with those at the top 20% or 40%, respectively, and run regressions with a binary treatment variable.

### 3.1 Assessment of the identification strategy

**Figure 3:**
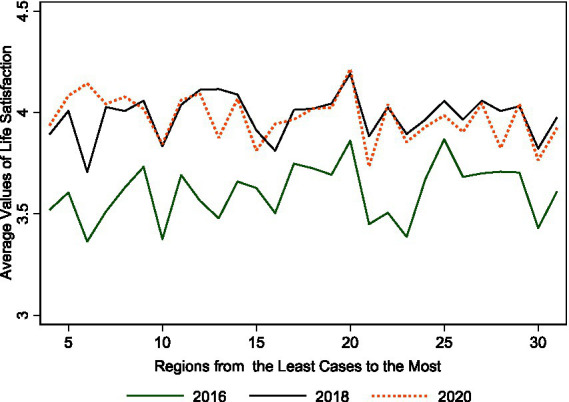
Trends of life satisfaction over regions with different exposure severity and time. We graphed regional average life satisfaction in Figure 3, ranging from the region with the lowest number of cases to the region with the highest number of cases for each year. We did not find life satisfaction decreasing with cases increasing.

The underlying assumption for the DiD estimator is that, in the absence of the pandemic, subjective wellbeing among the older adult exhibits parallel trends over regions and the pattern changes because of variations in exposure severity. This assumption ensures that the decline in life satisfaction is not driven by systematic differences across regions. We could not observe the counterfactual outcomes without the pandemic and therefore we tested the assumption directly. Providing a graphical depiction of the identification strategy is complicated in our context with individual life satisfaction as the outcome. Spatial and temporal variations in treatment intensity with three waves of longitudinal data lead to more difficult analyses, unlike the usual difference-in-differences setting with a binary treatment variable and data that include a greater number of time frequencies. We thereby offered a graphical illustration of the basic idea of our identification strategy in the spirit of an event study. We first graphed regional average life satisfaction in Figure 3, ranging from the region with the lowest number of cases to the region with the highest number of cases for each year. There is, approximately, a pattern of lines for 2016 and 2018 that is close to parallel trends over regions, while the year 2020 exhibits another pattern. Life satisfaction levels in those with severe exposure are slightly lower than in 2018. Similar patterns were found when we graphed regional average satisfaction over 5 or 10 quartile measures (see Supplementary Figure 1).

Second, we tested the validity of the identification strategy by challenging the possibility that the effects captured might stem from systematic regional differences. We run regressions of life satisfaction on groups of dummies indicating varying *exposure intensity* categories while controlling for wave, birth cohort, and individual fixed effects with 2016–2018 and 2018–2020 panels. Intuitively, the patterns of coefficients should differ when using data with and without the pandemic. As shown in column (1) of Supplementary Table 4, the coefficients of interest are all nonsignificant and suggest that there were no differences across regions grouped by exposure severity before the pandemic. For the 2018–2020 fixed effects estimation (see column 2), however, almost all of the coefficients are significant and their magnitudes suggest a declining trend in life satisfaction along with severity categories, although the coefficient of the most severely infected group, individuals residing in Hubei province, is insignificant. We also assigned the treated time to the year 2018 and run placebo tests with the 2016 and 2018 CFPS waves. Empirically, we used all respondents aged over 60 years to conduct cross-sectional analyses as well as run fixed effects estimation while controlling for the battery of variables listed in Equation 1. The counterfactual DiD estimators, as expected, are insignificant.

## 4 Empirical findings

### 4.1 Main results

Table 2 presents our main results showing severely-treated effects of COVID-19 on life satisfaction among the older adult. Three groups of regressions are presented, containing the whole panel, the balanced panel with respondents followed for all 3 years, and a cross-sectional study with all respondents aged 60 years and above. Each column represents a single regression. The results reported in column (1) reflect a short regression while controlling for wave and individual fixed effects, while the results reported in column (2) reflect further controls for birth cohort and province fixed effects. Column (3) presents estimates in the regression controlling for covariates, time and individual fixed effects. Column (4) presents the full estimates of Equation 1 with all individual socioeconomic and demographic variables added. Columns (5) and (6) display estimates from cross-sectional analyses. For columns (7) and (8), we re-estimated Equation 1 with the well-balanced panel.

**Table 2:** Intensive exposure to pandemic and associated policy and life satisfaction—panel results.

	Life satisfaction							
Data	Whole panel				Cross section		Balanced panel	
	(1)	(2)	(3)	(4)	(5)	(6)	(7)	(8)
Interaction	−0.0341***	−0.0341***	−0.0357***	−0.0366***	−0.0395***	−0.0344***	−0.0322***	−0.0340***
	(0.011)	(0.011)	(0.011)	(0.011)	(0.009)	(0.009)	(0.011)	(0.011)
Exposure Severity	0.0365	−0.0682	−0.003	−0.242	0.736***	0.734***	0.0582	0.0189
	(0.060)	(0.480)	(0.063)	(0.498)	(0.037)	(0.034)	(0.068)	(0.494)
coronavirus year	0.540***	0.542***	0.606***	0.715***	0.571***	0.678***	0.539***	0.776***
	(0.037)	(0.037)	(0.113)	(0.153)	(0.033)	(0.092)	(0.039)	(0.161)
Year = 2018	0.374***	0.377***	0.380***	0.436***	0.401***	0.425***	0.368***	0.465***
	(0.015)	(0.015)	(0.055)	(0.075)	(0.014)	(0.045)	(0.018)	(0.079)
Constant	3.752***	3.766**	7.158***	9.026***	2.088***	8.313***	3.689***	9.716***
	(0.182)	(1.749)	(2.129)	(3.303)	(0.0672)	(2.134)	(0.202)	(3.196)
Controls	NO	NO	Yes	Yes	NO	Yes	NO	Yes
Birth Cohort FE	NO	Yes	NO	Yes	Yes	Yes	No	Yes
Province FE	NO	Yes	NO	Yes	Yes	Yes	No	Yes
Wave FE	Yes	Yes	Yes	Yes	Yes	Yes	Yes	Yes
Individual FE	Yes	Yes	Yes	Yes	NO	NO	Yes	Yes
Observations	17,281	17,246	16,724	16,701	20,894	20,336	11,955	11,697
*R*-squared	0.596	0.598	0.616	0.619	0.063	0.173	0.575	0.601

All estimates are obtained with high dimension FE models; Respondents are respondents at ages of 60 and above from 2016 to 2020 waves of CFPS; Controls include gender, age, rural or urban residential, personal social status, marital status, and education attainment; Robust standard errors are reported in parentheses, while standard errors are adjusted for clusters in individuals; Robust standard errors have been alternatively clustered to provincial as well as personal level and the results are consistent; ***, **, and *, respectively, indicate significance levels of 1, 5, and 10%.

Our regressor of interest, *Interaction*, is consistently negative and significant across all regressions. This finding suggests that the older adult who reside in severe exposure areas evaluate their life satisfaction to be significantly lower than those experiencing less severe exposure. The sizes of the estimates of interest rise slightly when we controlled for age, gender, residential area, education, social status, and marital status, changing from −0.0341 to −0.0366. The average value of *severity* in 2020 is 2.93, and the average reduction in life satisfaction is estimated to be 0.11 (−0.0366*2.93) on the 5-point scale and around 3% of the regression sample average (−0.11/4.2; see Supplementary Table 3). The DiD estimate of cross-sectional studies after controlling for the battery of control variables is −0.0344, while it is −0.034 for the full estimation using the well-balanced panel sample. The effect sizes in the full estimations continue to be quite close, no matter which sample or method we used (see columns 4, 6, and 8). Therefore, the evidence strongly supports the proposition that there exists a negative impact of living in regions with more severe infections and strict distancing policies on wellbeing among the older adult. Note that the negative DiD estimates remain strongly significant in specifications without provincial fixed effects, birth-cohort fixed effects, or neither, and in estimations with robust standard error clustered by province or individual.

### 4.2 Results from alternative identification strategies

As discussed in section 3, we constructed alternative measures to capture regional severity of exposure. We re-estimated the difference-in-differences estimations with these alternative measures and presented the results in Table 3. In panel A, we continued to identify exposure severity with infections but in different ways. For columns 1 and 2 we used the usual difference-in-differences setting, comparing the bottom 20% or 40% of infected regions with the top 20% or 40% of severely exposed groups. The DiD estimates are significant and the coefficient rises to a greater extent (−0.130 for the top and bottom 20% groups by comparison; −0.126 for the top and bottom 40% groups by comparison). The variables containing the 2, 5, and 10 quartile categories of infections are then used instead of *exposure severity* in columns 3 through 5, while the natural log of regional infection cases is used in column 6. The last column presents the DiD estimator with the 2018–2020 panel. All DiD estimates remain strongly and significantly negative, except for changes in magnitude.

**Table 3:** Robustness checks with alternative identification strategies.

	Life satisfaction						
Panel A	Identify severity through cumulative infection cases						
Strategies	Top and bottom		2 quartiles dummy	5 quartiles severity	10 quartiles severity	Log(cases)	Interaction
	20%	40%					2018 and 2020
DiD estimate	−0.130**	−0.126***	−0.0763**	−0.0399***	−0.0194***	−0.0448***	−0.0262**
	(0.052)	(0.035)	(0.031)	(0.011)	(0.005)	(0.016)	(0.011)
Observations	6,446	13,322	16,701	16,701	16,701	16,701	7,832
*R*-squared	0.618	0.620	0.619	0.619	0.619	0.619	0.690
Panel B	Identify severity through number of deaths						
Strategies	Top and bottom 20%	Top and bottom 40%	2 quartiles dummy	5 quartiles severity	10 quartiles severity	Log (deaths)	Interaction 2018 and 2020
DiD estimate	−0.141***	−0.116***	−0.106***	−0.0349***	−0.0188***	−0.0249*	−0.0262**
	(0.046)	(0.035)	(0.031)	(0.010)	(0.0052)	(0.013)	(0.011)
Observations	9,428	13,304	16,701	16,701	16,701	15,576	7,832
*R*-squared	0.606	0.619	0.619	0.619	0.619	0.621	0.690
Panel C	Samples excluded Hubei province						
Strategies	Interaction	2 quartiles infection cases	5 quartiles infection cases	10 quartiles infection cases	Log(cases)	Log(deaths)	Interaction 2018 and 2020
DiD estimate	−0.0411***	−0.0809***	−0.0427***	−0.0211***	−0.0744***	−0.0608***	−0.0320***
	(0.011)	(0.031)	(0.011)	(0.006)	(0.019)	(0.019)	(0.012)
Observations	16,444	16,444	16,444	16,444	16,444	15,319	7,720
*R*-squared	0.619	0.619	0.619	0.619	0.619	0.620	0.689

All estimates are obtained with high dimension FE models; Respondents are respondents at ages of 60 and above; Controls are the same as full regressions in Table 2. Standard errors are reported in parentheses; Robust standard errors have been alternatively clustered to provincial as well as personal level and the results are consistent; ***, **, and *, respectively, indicate significance levels of 1, 5, and 10%.

For panel B, we identified exposure severity through regional numbers of deaths instead of infections and follow the same strategies. All estimates of interest are strongly and significantly negative. Nevertheless, the effect sizes are similar to those of their counterparts reported in panel A. In panel C, we considered samples that exclude Hubei province. As stated in section 2, the first wave of a major outbreak of COVID-19 in China occurred in Wuhan city, Hubei province, and the number of infections as well as deaths exceeds the sum of all infections and deaths in the remaining regions. The impacts of the pandemic on residents in Hubei province can be complex. After excluding Hubei province from the sample, all the DiD estimates remain strongly significant and rise compared with estimates obtained that include Hubei (for example*, interaction,* −0.0366 vs. –0.0411 for the 2016–2020 panel; −0.032 vs. –0.026 for the 2018–2020 panel). This evidence implicitly suggests that the loss in welfare is more likely to be related to health-risk perceptions and lockdown policies than to the infectious disease.

We then examined the relationship with dummies of *interaction* categories instead of one DiD item (see Supplementary Tables 5, 6). The decreasing patterns and significances of the coefficients from the lower to the higher exposure groups, in general, support the loss in welfare reflecting severe exposure. The insignificances of the counterfactual DiD estimates with the 2016 and 2018 waves, as placebo tests, also support the presence of a causal relationship (see Supplementary Table 4). In addition, we narrowed our focus to Hubei respondents alone. We found within-person increases in loneliness and pessimism (feeling that it is difficult to accomplish tasks) from 2018 to 2020 but no changes in life satisfaction (see Supplementary Table 7). This province experienced an extremely severe outbreak of disease infection and deaths compared with what other provinces experienced. Hubei residents have not, however, exhibited the greatest loss in subjective wellbeing, instead exhibiting resilience.

In general, the results reported in Table 3 are notable for their robustness to alternative identification strategies and subsamples, highlighting the negative impacts of severe exposure to the pandemic on life satisfaction among the surviving Chinese older adult. These results also imply that the overall negative effects are not sensitive to variations in identification strategies or sample selection.

### 4.3 Mechanism exploration

#### 4.3.1 Physical and mental health mechanisms

This section explores mechanisms that may explain the negative association found above. We focused on channels related to physical and mental health outcomes, through which severe exposure to COVID-19 as well as isolation affects subjective wellbeing among the older adult. In particular, we explored whether severe exposure to the pandemic is associated with the mechanisms in question by estimating:


(2) $$\begin{array}{l} \mathrm{mechanis}m_{iot}=\alpha +\lambda_{i}+\beta_{1}\mathrm{Interactio}n_{iot}\\ +\beta_{2}\mathrm{Exposure}\;\mathrm{Intensit}y_{\mathrm{io}}\\ +\beta_{3}\mathrm{coronavirus}\;year_{\mathrm{it}}\\ +\emptyset_{t}+\delta_{o}+\psi_{j}+\varepsilon_{\mathrm{it}} \end{array}$$

All estimation results are presented in Table 4 and for brevity we reported only the results for the key estimators (other results are available upon request). Variables examined include self-rated health condition, smoking behavior, chronic disease and sickness, sleeping difficulties, frequencies of various emotions (e.g., loneliness, distress, etc.). Empirically, for a mechanism to be capable of explaining the relationship between severe exposure to the pandemic and life satisfaction among the older adult, the $\beta_{1}$ estimates are expected to be statistically significant as a sufficient condition.

**Table 4:** Explore the mechanisms of health outcomes.

Panel A							
Dependent variable	Health status	Chronic disease	Sickness	Smoking	Feel hard to do everything	Sleeping difficulties	Loneliness
Interaction	0.0154	0.0106**	0.0115**	−0.001	0.0197*	0.0191*	−0.007
	(0.011)	(0.005)	(0.005)	(0.003)	(0.011)	(0.011)	(0.009)
coronavirus year	−0.022	−0.070***	−0.114***	−0.0192**	0.0898**	0.0091	0.121***
	(0.036)	(0.016)	(0.016)	(0.009)	(0.039)	(0.036)	(0.032)
Exposure Severity	−0.709	−0.310	0.0481	−0.105	0.213	0.471	−0.034
	(0.504)	(0.218)	(0.230)	(0.113)	(0.494)	(0.465)	(0.404)
Observations	19,946	19,956	19,956	17,264	17,188	17,257	17,191
*R*-squared	0.675	0.544	0.561	0.895	0.581	0.660	0.598

Equation 2 is estimated for all the possible outcomes in Table 4.

The results show that severe exposure to COVID-19 and related policies significantly increased the probability that chronic disease occurs in the previous half year as well as being ill during the previous 2 weeks. Smoking behaviors diminished in 2020 but the DiD estimate is insignificant. Poor health is negatively related to life satisfaction (42, 43). Moreover, Grimes (13) find that lockdown policies in New Zealand intensify feelings of loneliness. We found no significant effect, however, of severe exposure on loneliness among the older adult in fixed effects estimations. This might reflect the difficulty involved in confirming causality with a cross-sectional design. Moreover, this inconsistency might be driven by lifestyle differences in China (large families) and increases in online social interactions. During the pandemic, a health quick-response code was launched across the country and this policy promoted considerable coverage through internet and social media usage among the older adult. The percentage of internet users aged 60 years and over increased from 6.7% in 2019 to 11.2% in 2020. At the meantime, we observed that, on average, the level of loneliness increased significantly during the COVID-19 period compared with what occurred in previous waves (0.121). Furthermore, the results show that older adult individuals living in severe-exposure areas experienced greater difficulty falling asleep (0.02) and more frequently find it difficult to accomplish tasks (0.02) than their counterparts in areas with looser pandemic restrictions. It is possible that, in stricter regions, daily news about the pandemic and lockdowns promoted more prominent risk perceptions that are directly linked to psychological distress. Also, while social isolation is the major factor that is detrimental to wellbeing among the older adult and significantly associated with depression as well as loneliness [e.g., (44, 45)], mobility restrictions during the pandemic further exacerbated feelings of isolation.

In addition, we also investigated the influence of being overweight (with a BMI above 25), self-reported health status, and various emotions (the frequency of feeling happy, dismayed, sad, or pessimistic). See the Supplementary material for additional results. Both coefficients of *Interaction* and COVID-19 year are insignificant in regressions of overweight and self-reported health status as outcomes. The coefficients of COVID-19 year show increases in passive emotions but no significant effects are found to have been caused by severe exposure to the pandemic. The coefficients of *interaction* are insignificant for these outcomes. To summarize these findings, the evidence suggests that exposure to COVID-19 has, to some extent, generated negative influences on mental and physical health (chronic disease, illness, feeling that it is difficult to accomplish tasks, and sleeping difficulties), which are important determinants of subjective wellbeing, among the older adult.

#### 4.3.2 Robustness checks on the mechanisms

Because there was no major public health event between 2016 and 2018 that could affect health outcomes for the older adult as severely as the pandemic, we created an alternative *Interaction* term between exposure severity and 2018 and conduct placebo tests through estimating Equation 1. The health outcomes should not be influenced in such a quasi-counterfactual scenario and the coefficients of the alternatives should be insignificant, unlike the results presented in Table 4. As presented in Panel A of Table 5, as expected, all estimates of interest are statistically insignificant, supporting the results shown in Table 4.

**Table 5:** Robustness for the mechanisms.

Panel A	Placebo tests 2016–2018			
	Chronic Disease	Sickness	Feel hard to do everything	Sleeping difficulties
Exposure severity *Year = 2018	*0.001*	*−0.005*	*0.0012*	*0.0125*
	*(0.005)*	*(0.005)*	*(0.011)*	*(0.010)*
Exposure severity	0.0118	−0.125	−0.668	0.654
	(0.300)	(0.316)	(0.542)	(0.516)
Year = 2018	0.0265	0.0613***	0.107***	0.0213
	(0.0172)	(0.0181)	(0.0360)	(0.0342)
Constant	0.00434	1.037	5.157***	0.0583
	(1.015)	(1.069)	(1.817)	(1.731)
Observations	12,356	12,356	11,820	11,854
*R*-squared	0.626	0.636	0.657	0.722

Panel B	Life satisfaction			
	Cross sectional analyses		Panel study	
Interaction	−0.0346***	−0.0341***	−0.0303***	−0.0308***
	(0.011)	(0.011)	(0.009)	(0.009)
Chronic disease	0.023	0.022	−0.002	−0.0023
	(0.020)	(0.020)	(0.015)	(0.015)
Sickness	−0.0306	−0.0287	−0.0694***	−0.0602***
	(0.020)	(0.020)	(0.014)	(0.014)
Feel hard to do everything	−0.0413***	−0.0344***	−0.0924***	−0.0708***
	(0.010)	(0.010)	(0.008)	(0.008)
Sleeping difficulties	−0.009	−0.003	−0.0496***	−0.0356***
	(0.011)	(0.011)	(0.007)	(0.007)
Loneliness		−0.0467***		−0.112***
		(0.012)		(0.010)
Smoking		0.0317		−0.0531***
		(0.042)		(0.018)
Observations	16,638	16,600	20,284	20,249
*R*-squared	0.620	0.621	0.189	0.197

For cross-sectional analyses, other controls include social status, education, marital status, urban, gender, age, birth cohort fixed effect, province fixed effect and time fixed effect. Robust standard errors clustered to personal level are reported in parentheses; Robust standard errors have been alternatively clustered to provincial as well as personal level and the results are consistent; ***p < 0.01, **p < 0.05, *p < 0.1. The italicized values represent the regression coefficient, β1, which is the estimated interaction effect between the exposure severity variable and the 2018 year dummy, and indicates the treatment effect for the single year of 2018.

We further investigated the role of the mechanisms in explaining how the pandemic affects life satisfaction. To do this, we estimated the following:


(3) $$\begin{array}{l} \mathrm{life}\;\mathrm{satisfactio}n_{iot}=\alpha +\lambda_{i}+\mathrm{\gamma mechanis}m_{iot}\\ +\beta_{1}\mathrm{Interactio}n_{iot}\\ +\beta_{2}\mathrm{Exposure}\;\mathrm{Intensit}y_{\mathrm{io}}\\ +\beta_{3}\mathrm{coronavirus}\;year_{\mathrm{it}}\\ +\varphi X_{\mathrm{it}}+\emptyset_{t}+\delta_{o}+\psi_{j}+\varepsilon_{\mathrm{it}} \end{array}$$

Changes in $\beta_{1}$ of Equation (3) after controlling for the mechanism variables will help to explain the power of the mechanisms. Such a sequential covariate method of analysis has been used often in empirical studies to reveal mechanisms [see (46)]. The results are presented in Panel B of Table 5. In addition to fixed-effects estimations, cross-sectional analyses are also conducted.

Comparing the coefficients of Interaction before and after controlling for channel variables, the sizes of the estimates in the panel study decline from −0.0366 to −0.0341, accounting for 7% of the overall negative effect, while in the cross-sectional analysis the effects shrink from −0.0344 to −0.0308, accounting for 10.5% of the loss in satisfaction. This interpretation may suffer from unobserved collinearity, but it still provides us with a better understanding of the mechanisms. The determinants of life satisfaction are diverse and the pandemic with the associated quarantine policies have affected the older adult in complex ways. On the whole, both sets of estimates associated with Tables 4, 5 imply that COVID-19 exposure reduces overall life satisfaction among the older adult, while physical and mental health outcomes are significant channels for these effects.

### 4.4 Examination of heterogenous effects

The same external shock may have impact distinct groups differentially. Serrano-Alarcón et al. (47) find that mental health problems have been more serious among those with low educational attainment during the pandemic. Adams-Prassl et al. (4) point out that the negative impact of the pandemic on mental health has been attributable mainly to women, which is in line with Pierce et al. (48) and Bau et al. (49). Grimes (13) shows that individuals without partners experienced lower life satisfaction and greater loneliness. Moreover, Mahmud and Riley (50) find that the epidemic has had a considerable effect on the wellbeing of residents in rural areas. To identify which groups are more vulnerable, we further examined the older adult with alternative characteristics. This identification may also provide more targeted information about older adult sufferers to policymakers.

**Figure 4:**
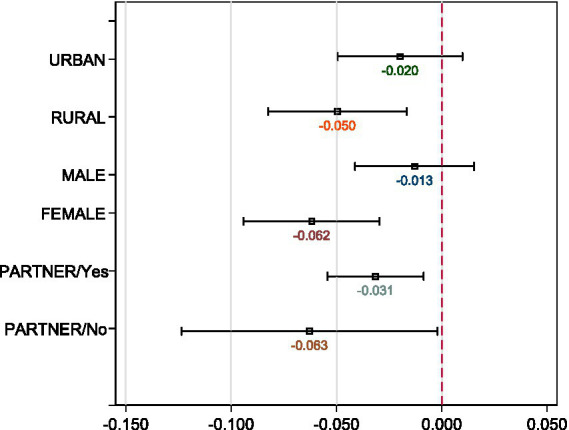
Heterogeneouse effects on life satisfaction across gender, residentials, and marital status. The DiD estimates of *Interaction* obtained with Equation 1 are graphed, respectively. All controls are the same as the full regressions in Table 2.

**Figure 5:**
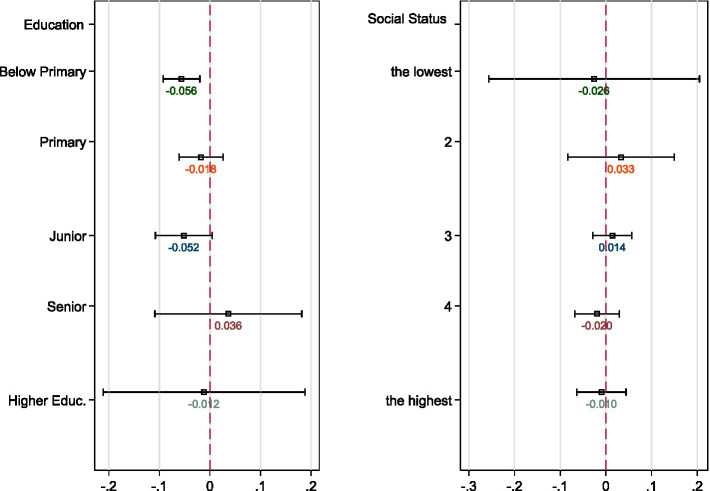
Heterogeneouse effects on life satisfaction across education attainment and social status. The DiD estimates of *Interaction* obtained with Equation 1 are graphed, respectively. All controls are the same as the full regressions in Table 2 but without the variable for classification.

We first estimated Equations 1, 2 across gender, rural–urban residents, and marital status (see Figures 4, 5).^5^ Regarding gender difference, we found females have experienced more significant decline in life satisfaction and more significant increases in sleeping difficulties as well as pessimistic moods than males. This is in line with Galasso et al. (51), who show that women are more likely to perceive the COVID-19 as a very serious health problem as well as being more likely to comply with public policy measures. Regarding smoking behavior, the estimates of the COVID-19 year dummy reveal reduced smoking among both males and females. The coefficients of *Interaction* are not consistent across gender. The estimate is negative for males while it is positive and marginally significant at the 10% level for females. Compared with individuals with partners, those without spouses have suffered twice the loss of life satisfaction during the pandemic, indicating that those without partners have been more vulnerable during the pandemic. These individuals have been more likely to experience illness and to encounter greater sleeping difficulties during the pandemic and lockdowns.

**Figure 6:**
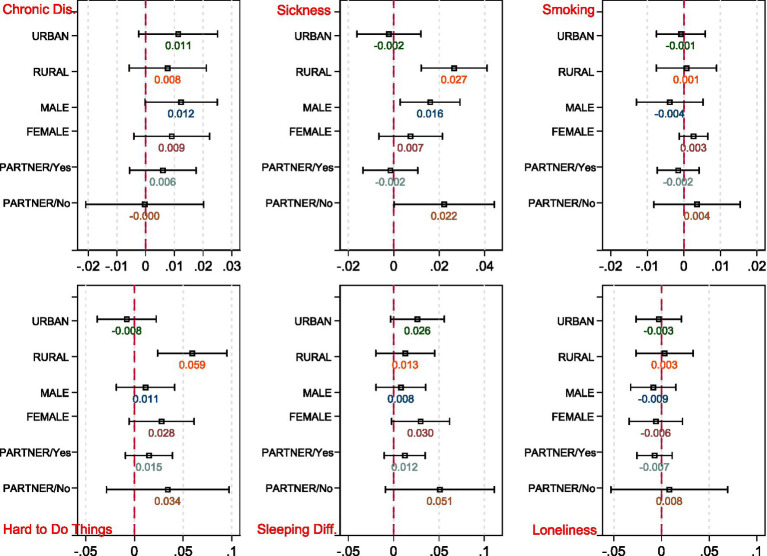
Heterogeneouse effects on health outcomes across gender, residentials, and marital status. The DiD estimates of *Interaction* obtained with Equation 2 are graphed, respectively.

Moreover, older adults living in rural areas suffer more severely than the urban older adult (see Figure 6). In general, most rural areas experience relatively worse socio-economic environments and healthcare conditions, with less developed digitalization. Moreover, family and social ties are arguably stronger in rural China than in urban areas. Thus, social isolation largely reduces social contacts among the rural older adult. We observed a significantly stronger reduction in the level of life satisfaction among the rural older adult. In severely exposed regions, older rural adults have experienced a significant increase in pessimistic moods and illness. In addition, we found that the urban older adult encounter more severe sleeping concerns caused by the pandemic than the rural older adult.

The heterogeneous effects on life satisfaction across educational level and social status are shown in Table 6. The estimates suggest that severe exposure to the pandemic is more detrimental to the older adult with lower educational attainment (below primary or junior high school), which is consistent with Serrano-Alarcón et al. (47). Less educated older adults have been significantly affected by the pandemic, reporting lower life satisfaction as well as a higher probability of suffering pessimistic moods and chronic disease during the previous half year (see estimation results reported in Supplementary Tables 7, 8). However, no significant heterogeneous effects are observed across social status. Based on the above analyses, individuals in the older adult population who are female, living alone, living in rural areas, or have lower educational attainment experience greater loss of wellbeing during the pandemic, and also report lower life satisfaction and worse physical or mental health.

**Table 6:** The heterogeneous effects over life cycle.

	Life satisfaction				
Age spans	≤20	20–40	40–60	≥60	≥65
Interaction	−0.0245	−0.0268***	−0.0165*	−0.0366***	−0.0388***
	(0.025)	(0.009)	(0.009)	(0.011)	(0.014)
Exposure severity	0.223*	−0.0283	0.547**	−0.242	2.246*
	(0.124)	(0.127)	(0.272)	(0.498)	(1.192)
Coronavirus year	−0.899***	0.382***	0.373***	0.715***	0.437**
	(0.336)	(0.0964)	(0.127)	(0.153)	(0.209)
Year =2018	−0.377**	0.318***	0.337***	0.436***	0.278***
	(0.159)	(0.0449)	(0.0615)	(0.0748)	(0.102)
Constant	−0.168	3.732***	1.900	9.026***	0.497
	(1.568)	(0.859)	(1.876)	(3.303)	(4.490)
Observations	2,726	21,120	26,085	16,701	10,067
*R*-squared	0.637	0.639	0.635	0.619	0.616

Same controls as the full regressions in Table 2. Robust standard errors are reported in parentheses; Robust standard errors have been alternatively clustered to provincial as well as personal level and the results are consistent; ***, **, and *, respectively, indicate significance levels of 1, 5, and 10%.

Last, we investigated whether older adult individuals are more vulnerable than younger cohorts during the pandemic. We applied Equation 1 for a range of age-cohort groups: below 20 years of age, those aged 20–40, those aged 40–60, and those aged 60 and above. Table 6 shows the results. As the sizes and significance of the coefficients of *Interaction* show, the pandemic has affected the older adult to the greatest extent (the coefficient is −0.036 for those aged 60 years and above, −0.04 for those aged 65 years and above), followed those aged 20–40 years (−0.029) and middle-age cohorts aged 40–60 years (−0.017). For those who are younger than 20 years of age, no significant effect is found. We applied permutation tests with 1,000 repetitions for the older adult population and other age groups. The resulting coefficient of *Interaction* for the older adult is significantly different from those for the other groups, although it is insignificant compared with those 25–35/20–40 years old. Thus, in contrast to the results for various stages of the life cycle, the negative effect of the pandemic on the older adult is the largest, suggesting that the pandemic and the associated lockdowns have affected wellbeing among the older adult the most severely.

## 5 Conclusion

The contemporary human society is confronted with a substantial challenge: population aging (52). In 2021, China had a population of approximately 267.36 million individuals aged 60 years and older, representing 18.9% of the national population, and 201 million individuals aged 65 years and older, accounting for 14.2% of the population. The improvement of the wellbeing of the older adult is an important aspect of the enhancement of social welfare (53, 54). However, the recent outbreak of COVID-19 has had a profound impact on the health and wellbeing of older adults (55). The associated large-scale lockdowns have distinguished this public health emergency from any previous pandemic. Both health risk and social isolation are widely documented as the two major determinants of wellbeing among the older adult (42). Consequently, it is of great significance to understand the effect and mechanisms of this public health emergency on wellbeing among the older adult (56, 57). This paper empirically answered the above questions with advanced research designs based on longitudinal individual-level data from China for 2016 through 2020.

The DiD estimators with the first-wave eruption of the COVID-19 pandemic in China as the treatment indicated that, first, life satisfaction among older populations has been negatively affected by the pandemic and lockdowns, conditional on year and individual fixed effects. Older populations in regions that were subject to wider virus spread and stricter social distancing has experienced a significant reduction in life satisfaction in 2020 after the first-wave outbreak in China, compared with what they experienced in 2016 and 2018. This suggests a causal relationship between isolation and the wellbeing of older adults. When examining various stages of the life cycle, it was evident that the older population was the most severely affected group. In light of the growing proportion of the global population that is aged 60 and over, the impact of containment measures on wellbeing is a matter of considerable concern.

Second, after performing a battery of identification strategy and specification checks, we explored several channels through which the pandemic has affected wellbeing among the older adult. Our results show that such exposure has increased the probability of suffering from chronic disease in the preceding half year as well as illness during the preceding 2 weeks. With respect to depression, the older adult living in areas subject to severe exposure has experienced greater difficulty falling asleep and more frequently considered it difficult to accomplish daily tasks. Furthermore, the coefficients of the COVID year reflect a decrease in smoking behavior and increases in various passive emotions, but no stronger effects are found to have been caused by extensive exposure to the pandemic because of the insignificance of the DiD estimators.

Third, we found heterogeneity in the effects of lockdowns across various groups. Regarding gender, the pandemic has led to a significant decline in life satisfaction and higher frequency of sleeping difficulties as well as pessimistic moods among females than among males. Regarding living status, populations without spouses have suffered loss of life satisfaction that was two times greater than that experienced by married individuals during the pandemic. Moreover, older adult individuals living in rural areas have suffered more severe consequences than the urban older adult. We observed an average increase in loneliness in the year of the pandemic in the rural sample but not in the urban sample. We also discovered that the urban older adult has encountered more severe sleep problems during the pandemic than the rural older adult. In addition, older adult individuals with the lowest educational attainment level (below primary schooling) are more vulnerable and we also found that those with zero income have suffered from significantly more severe loneliness during the pandemic.

Our findings not only add new evidence to a growing literature that examines various consequences of the pandemic, but also reveal insights that have significant implications for public policies. When policies are designed to prevent viral spread and protect public health, it is important to consider the prevention of secondary disasters, such as poorer physical and mental health among the older adult. First, it is recommended that psychological counseling be provided to the older adult, and counseling that is appropriate to various age groups and older adults across socio-economic backgrounds should be considered. This suggestion is also supported by Coyle and Dugan (30), who find that older individuals who can endure social isolation or adjust their expectations so that they do not feel subjective isolation may experience better physical and mental health. Second, elder-oriented policies, especially policies that focus on mental disturbance regulations and avoid perceived social isolation, should be designed to help individuals overcome the negative influences of the pandemic and concurrent lockdown measures. These policies could promote digital inclusion and institute special medical treatment tracks. Third, in Hubei province, where there has been a dramatic increase in infection and the highest number of deaths from COVID-19, there is no evidence to suggest that there has been a significant loss of life satisfaction. However, there is a negative influence on mental health. This finding implies that responses to public health emergencies should consider the significant cost of isolation measures in terms of wellbeing, especially among older adults.

In conclusion, it is important to acknowledge several limitations of the current study. Firstly, it is not possible to isolate the impact of the disease itself from that of social isolation and social distancing policies based on the data available at the time of analysis. Secondly, the estimates presented in this study are based on data from the early stages of the pandemic and, as a result, should be interpreted as representing the immediate health impacts of the pandemic and social isolation. Future studies may be able to distinguish the combined effects of infection and social isolation threats, or the long-term impact of the pandemic on mental health, when detailed policies data and more recent data become available.

## Data Availability

Publicly available datasets were analyzed in this study. This data can be found here: https://www.isss.pku.edu.cn/cfps/.

## Footnotes

^1^Measures and responses at Level I are organized and implemented by the State Council and by governments at the provincial level. Under the unified leadership and command of the State Council, measures include delineating control areas, instituting coercive measures, restricting mobility, controlling traffic, implementing health quarantines, etc. For Level II, all responses are the responsibility of governments at the provincial level. For Level III, municipal governments lead and direct emergency responses within their administrative areas. Governments at the county or local level organize and implement public health emergency measures at level IV. At any time, higher-level governments can give guidance and support to lower-level governments.

^2^“China’s economy continues to bounce back from virus slump.” BBC News. 19 October 2020. Retrieved 9 January 2021; “China’s economic recovery continues but signals mixed in October.” Nikkei Asia. Retrieved 9 January 2021. Information source: https://en.wikipedia.org/wiki/COVID-19_pandemic_in_mainland_China#2019-2020_outbreak

^3^The CFPS is conducted by the Institute of Social Science Survey (ISSS), funded by Peking University. Note that the questionnaires vary by year. From 2016 through 2020, most observations were the same but changes in the interviewee population change over time. The total number of adult respondents in the 2020 wave is approximately 26,400, less than the approximately 30,000 who had responded in previous waves.

^4^The original contributions presented in the study are publicly available. This data can be found here: sss.pku.edu.cn/cfps/en/index.htm

^4^The sample covers 27 provinces and four megacities. Eight areas are included in quantile 1 with the number of infections ranging from 1 to 224; six areas are included in quantile 2 with the number of infections ranging from 230 to 373; seven areas are categorized as quantile 3 with the number of infections ranging from 507 to 935; quantile 4 consists of six areas with the number of infections ranging from 964 to 1,299; quantile 5 contains three areas with the number of infections ranging from 1,306 to 2,046; Hubei province is included in quantile 6 and has 68,149 infected cases.

^5^Main regression results are presented in Supplementary Tables 9, 10 and additional results are available upon request.
