# Supplementary material for: Influences of public health emergency and social isolation on older adults’ wellbeing: evidence from a longitudinal study
Source: Front Public Health. 2024 Aug 29;12:1417610. doi: 10.3389/fpubh.2024.1417610 (PMC11395494; doi:10.3389/fpubh.2024.1417610)
Supplement: Supplementary file 1 [file Data_Sheet_1.docx]

**Appendix**

**Figure 1: Subjective Well-being Changes along 10 Quartile Measure of Severity Over Time.**

**Table 1: Response time from Level I to Level IV in different provinces.**

| **Province** | **Timing of Level I** | **Adjusted to Level II** | **Adjusted to Level III** | **Adjusted to Level IV** |
| --- | --- | --- | --- | --- |
| Beijing | Jan 24 | April 30 | Jun 5 |  |
| Tianjin | Jan 24 | April 30 | Jun 6 |  |
| Hebei | Jan 24 | April 30 | Jun 6 |  |
| Shanxi | Jan 25 | Feb 24 | Mar10 |  |
| Nei Mongol | Jan 25 |  | Feb 25 |  |
| Liaoning | Jan 25 |  | Feb 22 |  |
| Jilin | Jan 24 | Feb 26 | Mar 20 |  |
| Heilongjiang | Jan 25 | Mar 4 | Mar 25 |  |
| Shanghai | Jan 24 | Mar 24 | May 9 |  |
| Jiangsu | Jan 24 | Mar 24 | Mar 27 |  |
| Zhejiang | Jan 23 | Mar 2 | Mar 23 |  |
| Anhui | Jan 24 | Feb 25 | Mar 15 |  |
| Fujian | Jan 24 | Feb 26 | Feb 26 |  |
| Jiangxi | Jan 24 | Mar 12 | Mar 20 |  |
| Shandong | Jan 24 | Mar 7 | May 5 |  |
| Henan | Jan 25 | Mar 19 | May 5 |  |
| Hubei | Jan 24 | May 2 | Jun 13 |  |
| Hunan | Jan 23 | Mar 10 | Mar 31 |  |
| Guangdong | Jan 23 | Feb 24 | May 9 |  |
| Guangxi | Jan 24 |  | Feb 24 |  |
| Hainan | Jan 25 | Jan 24 | Feb 26 |  |
| Chongqing | Jan 24 | Mar 10 | Mar 24 |  |
| Sichuan | Jan 24 | Feb 26 | Mar 25 |  |
| Guizhou | Jan 24 |  | Feb 23 |  |
| Yunnan | Jan 24 |  | Feb 24 |  |
| Xizang | Jan 30 |  |  |  |
| Shan'xi | Jan 25 |  | Feb 28 |  |
| Gansu | Jan 25 |  | Feb 21 | May 11 |
| Qinghai | Jan 25 |  | Feb 26 | Mar 6 |
| Ningxia | Jan 25 | Feb 28 | May 6 |  |
| Xinjiang | Jan 25 | Feb 25 | Mar 7 | Mar 21 |

| **Table 2: Regional COVID-19 Cumulative Infected Cases and Deaths.** | | |
| --- | --- | --- |
| Province (2020) | COVID-19 Cases | Death |
| Beijing | 987 | 9 |
| Tianjin | 309 | 3 |
| Hebei Sheng | 373 | 6 |
| Shanxi Province | 224 | 0 |
| Nei Mongol | 364 | 1 |
| Liaoning Province | 351 | 2 |
| Jilin Province | 157 | 2 |
| Heilongjiang Province | 964 | 13 |
| Shanghai | 1516 | 7 |
| Jiangsu Province | 684 | 0 |
| Zhejiang Province | 1306 | 1 |
| Anhui Province | 993 | 6 |
| Fujian Province | 513 | 1 |
| Jiangxi Province | 935 | 1 |
| Shandong Province | 862 | 7 |
| Henan Province | 1299 | 22 |
| Hubei Province | 68149 | 4512 |
| Hunan Province | 1021 | 4 |
| Guangdong Province | 2046 | 8 |
| Guangxi | 264 | 2 |
| Hainan Province | 171 | 6 |
| Chongqing Shi | 590 | 6 |
| Sichuan Province | 853 | 3 |
| Guizhou Province | 147 | 2 |
| Yunnan Province | 230 | 2 |
| Tibet | 1 | 0 |
| Shaanxi Province | 507 | 3 |
| Gansu Province | 182 | 2 |
| Qinghai Province | 18 | 0 |
| Ningxia | 75 | 0 |
| Xinjiang | 980 | 3 |

| **Table 3: Statistics Description of Regression Sample.** | | | | | |
| --- | --- | --- | --- | --- | --- |
| Variable | **Obs.** | **Mean** | **Std. Dev.** | **Min** | **Max** |
| **Life Satisfaction: 1-5 from the lowest to the highest** | | | | | |
|  | 17281 | 4.119 | 0.976 | 1 | 5 |
| **Chronic Disease:** whether has got chronical disease during the past half year | | | | |  |
|  | 17281 | 0.393 | 0.488 | 0 | 1 |
| **Sickness:** whether is sick or not in the last two weeks | | | | | |
|  | 17281 | 0.400 | 0.490 | 0 | 1 |
| **Smoking:** whether smoked during the past month | | | | | |
|  | 17,278 | 0.281 | 0.449 | 0 | 1 |
| **Sleep Difficulties:** 1= never; 2= 1-2 days; 3= 3-4 days; 4= 5-7 days; frequencies per week | | | | | |
|  | 17273 | 1.919 | 1.030 | 1 | 4 |
| **Feel Hard to Do Everything:** 1= never; 2= 1-2 days; 3= 3-4 days; 4= 5-7 days; frequencies in a week | | | | | |
|  | 17233 | 1.885 | 0.985 | 1 | 4 |
| **Loneliness:** 1= never; 2= 1-2 days; 3= 3-4 days; 4= 5-7 days; frequencies in a week | | | | | |
|  | 17233 | 1.478 | 0.823 | 1 | 4 |
| **Social Status** related to wealth and income**: 1-5 from the lowest to the highest** | | | | | |
| 1 | 17168 | 0.080 | 0.271 | 0 | 1 |
| 2 | 17168 | 0.125 | 0.330 | 0 | 1 |
| 3 | 17168 | 0.387 | 0.487 | 0 | 1 |
| 4 | 17168 | 0.219 | 0.414 | 0 | 1 |
| 5 | 17168 | 0.189 | 0.392 | 0 | 1 |
| **Education Attainment: 1-5 from the lowest to the highest** | | |  |  |  |
| Below Primary | 17103 | 0.464 | 0.499 | 0 | 1 |
| Primary | 17103 | 0.237 | 0.425 | 0 | 1 |
| Junior High | 17103 | 0.185 | 0.388 | 0 | 1 |
| Senior High | 17103 | 0.089 | 0.285 | 0 | 1 |
| Higher education | 17103 | 0.017 | 0.129 | 0 | 1 |
| **Self-reported Health Status: 1-5 from the worst to the best** | | | | | |
| 1 | 17277 | 0.278 | 0.448 | 0 | 1 |
| 2 | 17277 | 0.195 | 0.396 | 0 | 1 |
| 3 | 17277 | 0.348 | 0.476 | 0 | 1 |
| 4 | 17277 | 0.099 | 0.298 | 0 | 1 |
| 5 | 17277 | 0.081 | 0.273 | 0 | 1 |
| **Marital Status** (1 Partner/Yes) | 17281 | 0.824 | 0.381 | 0 | 1 |
| **Male** | 17281 | 0.510 | 0.500 | 0 | 1 |
| **Urban** | 17224 | 0.480 | 0.500 | 0 | 1 |
| **Age** | 17281 | 68.130 | 5.869 | 60 | 95 |
| Note: regression sample based the first basic regression in table 2 | | | | | |

| **Table 4: Regression Tests for the Validity of Identification Strategy.** | | | | |  |
| --- | --- | --- | --- | --- | --- |
|  | **Life Satisfaction** | | | |  |
|  | (1) | (2) | (3) | (4) | (5) |
| Data | 2016&2018 | 2018&2020 | 2016&2018 | 2018&2020 | 2016&2018 |
| Post Year* Severity |  |  | -0.0161 | -0.0266*** | -0.0222 |
|  |  |  | (0.0174) | (0.00926) | (0.0227) |
| Severity |  |  | -0.263*** | 0.686*** | -0.453*** |
|  |  |  | (0.0239) | (0.0148) | (0.0568) |
| Post Year |  |  | 0.348*** | 0.105*** | 0.386*** |
|  |  |  | (0.0640) | (0.0224) | (0.0881) |
| Severity =2 | -0.0203 | 1.165*** |  |  |  |
|  | (0.625) | (0.448) |  |  |  |
| Severity =3 | 0.450 | 1.168*** |  |  |  |
|  | (0.549) | (0.442) |  |  |  |
| Severity =4 | 0.354 | 1.641*** |  |  |  |
|  | (0.617) | (0.525) |  |  |  |
| Severity =5 | 0.00119 | 0.821* |  |  |  |
|  | (0.529) | (0.434) |  |  |  |
| Severity =6 | 0.226 | 0.523 |  |  |  |
|  | (0.778) | (0.659) |  |  |  |
| Constant | 3.893*** | 3.294*** | 6.100*** | 0.848*** | 6.546*** |
|  | (0.404) | (0.307) | (0.123) | (0.114) | (0.729) |
| Observations | 11,832 | 7,984 | 15,420 | 12,678 | 11,334 |
| R-squared | 0.652 | 0.675 | 0.170 | 0.141 | 0.672 |

Note: Regression (1) and (2) are fixed effect estimations controlling birth cohorts, wave and individual fixed effects. Regression (3) and (4) are ordinary least square estimations and other controls include urban, gender, age, social status, education, marital status, birth cohort fixed effect, wave fixed effect and province fixed effect. Regression (5) are fixed effect estimation controlling for urban, gender, age, social status, education, marital status, birth cohort fixed effect, wave fixed effect, province and individual fixed effect. The robust standard errors are adjusted for clusters in provinces and are reported in parentheses. *** p<0.01, ** p<0.05, * p<0.1.

**Table 5: Effects of COVID-19 Exposure across Different Quantile Categories.**

| **Dependent Variable** | **Life Satisfaction** | | | | | | | | |
| --- | --- | --- | --- | --- | --- | --- | --- | --- | --- |
| method | Panel Study | | Cross Study | |  | Panel Study | | Cross Study | |
| waves | 16-20 | 18-20 | 16-20 | 18-20 |  | 16-20 | 18-20 | 16-20 | 18-20 |
| 5 Quantile |  |  |  |  | 10 Quantile |  |  |  |  |
| 1.interaction | 0.478*** | 0.129*** | 0.411*** | 0.0937*** | 1*year2020 | 0.493*** | 0.126*** | 0.433*** | 0.0925** |
|  | (0.0360) | (0.0365) | (0.0324) | (0.0338) |  | (0.0412) | (0.0424) | (0.0362) | (0.0382) |
| 2.interaction | 0.401*** | 0.0731** | 0.352*** | 0.0384 | 2*year2020 | 0.445*** | 0.151** | 0.319*** | 0.0740 |
|  | (0.0349) | (0.0347) | (0.0297) | (0.0298) |  | (0.0604) | (0.0617) | (0.0570) | (0.0604) |
| 3.interaction | 0.342*** | 0.0868** | 0.309*** | 0.0453 | 3*year2020 | 0.431*** | 0.0662 | 0.393*** | 0.0414 |
|  | (0.0360) | (0.0363) | (0.0300) | (0.0310) |  | (0.0443) | (0.0445) | (0.0375) | (0.0387) |
| 4.interaction | 0.281*** | -0.0117 | 0.226*** | -0.0474 | 4*year2020 | 0.332*** | 0.0544 | 0.300*** | 0.0387 |
|  | (0.0356) | (0.0359) | (0.0308) | (0.0319) |  | (0.0615) | (0.0628) | (0.0476) | (0.0489) |
| 5.interaction | 0.347*** | 0.0213 | 0.287*** | -0.0190 | 5*year2020 | 0.298*** | 0.0440 | 0.295*** | 0.0212 |
|  | (0.0381) | (0.0384) | (0.0322) | (0.0331) |  | (0.0464) | (0.0474) | (0.0383) | (0.0390) |
| 6.interaction | 0.416*** | 0.184 | 0.402*** | 0.154* | 6*year2020 | 0.405*** | 0.149*** | 0.331*** | 0.0839* |
|  | (0.130) | (0.135) | (0.0855) | (0.0856) |  | (0.0550) | (0.0566) | (0.0450) | (0.0507) |
|  |  |  |  |  | 7*year2020 | 0.282*** | -0.0117 | 0.226*** | -0.0471 |
|  |  |  |  |  |  | (0.0357) | (0.0360) | (0.0308) | (0.0319) |
|  |  |  |  |  | 9*year2020 | 0.398*** | 0.0710 | 0.315*** | 0.0335 |
|  |  |  |  |  |  | (0.0477) | (0.0490) | (0.0364) | (0.0381) |
|  |  |  |  |  | 10.year2020 | 0.267*** | -0.0591 | 0.246*** | -0.0968 |
|  |  |  |  |  |  | (0.0610) | (0.0619) | (0.0570) | (0.0594) |
| Constant | 4.393*** | 4.923*** | 6.005*** | 4.581*** |  | 4.334*** | 5.051*** | 6.010*** | 4.555*** |
|  | (1.207) | (0.625) | (0.125) | (0.130) |  | (1.275) | (0.677) | (0.125) | (0.131) |
| Observations | 16,783 | 7,890 | 20,032 | 12,494 |  | 16,522 | 7,774 | 20,032 | 12,494 |
| R-squared | 0.619 | 0.690 | 0.174 | 0.143 |  | 0.619 | 0.690 | 0.174 | 0.143 |
| Note: All controls in panel study are the same as Table 2. Cross-analyses control for urban, gender, social status, education, marital status, birth cohort fixed effect, wave fixed effect and province fixed effect. Empirical sample excludes Hubei province. Robust standard errors in parentheses; *** p<0.01, ** p<0.05, * p<0.1. | | | | | | | | | |

**Table 6: Effects of COVID-19 Exposure across Different Quantiles Excluded Hubei Sample.**

| **Dependent Variable** | **Life Satisfaction** | | | | | | | | |
| --- | --- | --- | --- | --- | --- | --- | --- | --- | --- |
| method | Panel Study | | Cross Study | |  | Panel Study | | Cross Study | |
| waves | 16-20 | 18-20 | 16-20 | 18-20 |  | 16-20 | 18-20 | 16-20 | 18-20 |
| 5 Quantile |  |  |  |  | 10 Quantile |  |  |  |  |
| 1.interaction | 0.480*** | 0.129*** | 0.412*** | 0.0938*** | 1*year2020 | 0.493*** | 0.126*** | 0.433*** | 0.0925** |
|  | (0.0361) | (0.0366) | (0.0324) | (0.0338) |  | (0.0412) | (0.0424) | (0.0362) | (0.0382) |
| 2.interaction | 0.403*** | 0.0732** | 0.353*** | 0.0386 | 2*year2020 | 0.445*** | 0.151** | 0.319*** | 0.0740 |
|  | (0.0350) | (0.0348) | (0.0298) | (0.0298) |  | (0.0604) | (0.0617) | (0.0570) | (0.0604) |
| 3.interaction | 0.342*** | 0.0871** | 0.310*** | 0.0457 | 3*year2020 | 0.431*** | 0.0662 | 0.393*** | 0.0414 |
|  | (0.0361) | (0.0364) | (0.0300) | (0.0310) |  | (0.0443) | (0.0445) | (0.0375) | (0.0387) |
| 4.interaction | 0.282*** | -0.0117 | 0.226*** | -0.0471 | 4*year2020 | 0.332*** | 0.0544 | 0.300*** | 0.0387 |
|  | (0.0357) | (0.0360) | (0.0308) | (0.0319) |  | (0.0615) | (0.0628) | (0.0476) | (0.0489) |
| 5.interaction | 0.349*** | 0.0210 | 0.288*** | -0.0189 | 5*year2020 | 0.298*** | 0.0440 | 0.295*** | 0.0212 |
|  | (0.0382) | (0.0385) | (0.0322) | (0.0332) |  | (0.0464) | (0.0474) | (0.0383) | (0.0390) |
|  |  |  |  |  | 6*year2020 | 0.405*** | 0.149*** | 0.331*** | 0.0839* |
|  |  |  |  |  |  | (0.0550) | (0.0566) | (0.0450) | (0.0507) |
|  |  |  |  |  | 7*year2020 | 0.282*** | -0.0117 | 0.226*** | -0.0471 |
|  |  |  |  |  |  | (0.0357) | (0.0360) | (0.0308) | (0.0319) |
|  |  |  |  |  | 9*year2020 | 0.398*** | 0.0710 | 0.315*** | 0.0335 |
|  |  |  |  |  |  | (0.0477) | (0.0490) | (0.0364) | (0.0381) |
|  |  |  |  |  | 10.year2020 | 0.267*** | -0.0591 | 0.246*** | -0.0968 |
|  |  |  |  |  |  | (0.0610) | (0.0619) | (0.0570) | (0.0594) |
| Constant | 5.565** | 1.860 | 6.005*** | 4.581*** |  | 5.378*** | 2.375 | 6.010*** | 4.555*** |
|  | (2.219) | (2.125) | (0.125) | (0.130) |  | (2.012) | (1.743) | (0.125) | (0.131) |
| Observations | 16,522 | 7,774 | 20,032 | 12,494 |  | 16,522 | 7,774 | 20,032 | 12,494 |
| R-squared | 0.618 | 0.689 | 0.174 | 0.143 |  | 0.619 | 0.690 | 0.174 | 0.143 |
| Note: All controls in panel study are the same as Table 2. Cross-analyses control for urban, gender, social status, education, marital status, birth cohort fixed effect, wave fixed effect and province fixed effect. Empirical sample excludes Hubei province. Robust standard errors in parentheses; *** p<0.01, ** p<0.05, * p<0.1. | | | | | | | | | |

| **Table 7: Evaluation of Other Outcomes.** | | | | | | | |
| --- | --- | --- | --- | --- | --- | --- | --- |
| Outcomes | Social Status | Quantities of Cigarette | Overweight  BMI>=25 | Frequency of  Feel Happy | Frequency of Feeling life cannot continue | Frequency of Feeling Sadness | For Most People: Trust or Doubt |
|  | (1) | (2) | (3) | (4) | (5) | (6) | (7) |
| **Interaction** | 0.0012 | -0.0087 | -0.004 | -0.0101 | -0.0023 | 0.0015 | -0.01 |
|  | (0.0130) | (0.153) | (0.00416) | (0.0116) | (0.00745) | (0.00859) | (0.00603) |
| Exposure Severity | -0.883 | -1.225 | 0.106 | -1.075** | 0.628* | 0.550 | 0.135 |
|  | (0.566) | (5.369) | (0.203) | (0.505) | (0.323) | (0.373) | (0.262) |
| coronavirus year | 0.446*** | -2.165*** | 0.0853** | -0.0374 | 0.0834*** | 0.0614** | 0.0969*** |
|  | (0.0441) | (0.508) | (0.0394) | (0.0394) | (0.0253) | (0.0291) | (0.0204) |
| Year=2018 | 0.344*** | -0.569*** | 0.0809** | -0.103*** | 0.0352*** | 0.0594*** | 0.00283 |
|  | (0.0172) | (0.209) | (0.0372) | (0.0154) | (0.00984) | (0.0113) | (0.00796) |
| Constant | 5.786*** | 21.41** | 0.414 | 6.348*** | -0.670 | -0.401 | 0.323 |
|  | (2.058) | (10.33) | (0.595) | (1.842) | (1.180) | (1.362) | (0.955) |
| Observations | 17,076 | 4,373 | 7,826 | 17,237 | 17,206 | 17,242 | 17,139 |
| R-squared | 0.601 | 0.806 | 0.834 | 0.563 | 0.570 | 0.615 | 0.537 |
| Note: Equation (2) with these outcomes is re-estimated. Robust standard errors clustered to personal level are reported in parentheses; *** p<0.01, ** p<0.05, * p<0.1. | | | | | | | |

| **Table 8: Examination of Heterogeneous Effects across Education Levels.** | | | | | | | | | | |
| --- | --- | --- | --- | --- | --- | --- | --- | --- | --- | --- |
| **Subsample** | **Below**  **Primary** | **Primary** | **Junior** | **Senior** | **Higher**  **Education** | **Below Primary** | **Primary** | **Junior** | **Senior** | **Higher**  **Education** |
| **Outcomes** | **Life Satisfaction** | | | | | **Self-reported Health Condition** | | | | |
| **Interaction** | -0.056*** | -0.0187 | -0.0219 | -0.0524* | 0.0113 | 0.0147 | 0.00450 | -0.0477** | -0.0371 | 0.0400 |
|  | (0.0184) | (0.0221) | (0.0217) | (0.0285) | (0.0569) | (0.0213) | (0.0246) | (0.0240) | (0.0301) | (0.0531) |
| coronavirus year | 0.533*** | 0.429*** | 0.499*** | 0.426*** | 0.191 | -0.0303 | -0.0107 | 0.131 | 0.231** | 0.0405 |
|  | (0.0601) | (0.0762) | (0.0781) | (0.104) | (0.215) | (0.0694) | (0.0845) | (0.0864) | (0.110) | (0.201) |
| Observations | 7,564 | 3,826 | 3,123 | 1,510 | 413 | 7,567 | 3,826 | 3,123 | 1,510 | 413 |
| R-squared | 0.597 | 0.632 | 0.659 | 0.660 | 0.616 | 0.674 | 0.707 | 0.718 | 0.730 | 0.731 |
| **Outcomes** | **Chronic Disease** | | | | | **Sick** | | | | |
| **Interaction** | 0.0149* | -0.00221 | 0.0103 | 0.00966 | -0.0122 | 0.00709 | 0.0103 | -0.00729 | 0.0206 | -0.0625** |
|  | (0.00875) | (0.0117) | (0.0117) | (0.0154) | (0.0357) | (0.00942) | (0.0120) | (0.0120) | (0.0154) | (0.0315) |
| coronavirus year | -0.0299 | 0.0331 | 0.00775 | -0.0515 | 0.0419 | -0.0576* | -0.0445 | 0.0535 | -0.130** | 0.210* |
|  | (0.0286) | (0.0402) | (0.0422) | (0.0564) | (0.135) | (0.0307) | (0.0414) | (0.0431) | (0.0563) | (0.119) |
| Observations | 7,572 | 3,826 | 3,124 | 1,510 | 413 | 7,572 | 3,826 | 3,124 | 1,510 | 413 |
| R-squared | 0.559 | 0.566 | 0.573 | 0.613 | 0.552 | 0.559 | 0.587 | 0.587 | 0.617 | 0.626 |
| **Outcomes** | **Feel Hard to do Things** | | | | | **Loneliness** | | | | |
| **Interaction** | 0.0490** | 0.0286 | -0.00874 | -0.0259 | -0.0392 | 0.00344 | -0.0114 | -0.0288 | 0.0122 | 0.0455 |
|  | (0.0197) | (0.0232) | (0.0232) | (0.0294) | (0.0551) | (0.0163) | (0.0191) | (0.0179) | (0.0216) | (0.0397) |
| coronavirus year | -0.0189 | 0.0408 | 0.251*** | 0.196* | 0.269 | 0.0696 | 0.127* | 0.170*** | 0.00486 | -0.0912 |
|  | (0.0640) | (0.0798) | (0.0835) | (0.108) | (0.208) | (0.0530) | (0.0658) | (0.0645) | (0.0789) | (0.150) |
| Observations | 7,525 | 3,815 | 3,122 | 1,509 | 412 | 7,533 | 3,816 | 3,124 | 1,507 | 413 |
| R-squared | 0.556 | 0.603 | 0.592 | 0.584 | 0.604 | 0.602 | 0.607 | 0.588 | 0.626 | 0.672 |
| Note: All controls are the same as full regressions in Table 2 but without education. Robust standard errors in parentheses; *** p<0.01, ** p<0.05, * p<0.1. | | | | | | | | | | |

| **Table 9: Examination of Heterogeneous Effects across Income Levels.** | | | | | | | | | |
| --- | --- | --- | --- | --- | --- | --- | --- | --- | --- |
| **Outcomes** | **Life Satisfaction** | | | **Chronic Disease** | | | **Sick** | | |
|  | **Missing** | **Income=0** | **Income>0** | **Missing** | **Income=0** | **Income>0** | **Missing** | **Income=0** | **Income>0** |
| **Interaction** | -0.0482*** | -0.243 | -0.0461 | 0.00971 | -0.0231 | 0.0269 | 0.00458 | -0.0276 | 0.0103 |
|  | (0.0138) | (0.372) | (0.0501) | (0.00701) | (0.171) | (0.0215) | (0.00724) | (0.163) | (0.0227) |
| Exposure Severity | 0.384 | 0.213 | 0.214 | -0.379 | 0.0349 | 0.887* | 0.236 | -0.489* | 0.872* |
|  | (0.555) | (0.652) | (1.115) | (0.286) | (0.297) | (0.479) | (0.296) | (0.284) | (0.505) |
| coronavirus year | 0.580*** | 1.194 | 0.623*** | 0.00527 | 0.314 | -0.0969 | -0.00951 | 0.290 | 0.00257 |
|  | (0.0470) | (0.914) | (0.196) | (0.0237) | (0.420) | (0.0844) | (0.0245) | (0.402) | (0.0891) |
| Observations | 8,763 | 975 | 731 | 9,175 | 977 | 731 | 9,175 | 977 | 731 |
| R-squared | 0.630 | 0.643 | 0.650 | 0.584 | 0.609 | 0.623 | 0.589 | 0.688 | 0.657 |
| **Outcomes** | **Smoking** | | | **Feel Hard to Do Things** | | | **Loneliness** | | |
|  | **Missing** | **Income=0** | **Income>0** | **Missing** | **Income=0** | **Income>0** | **Missing** | **Income=0** | **Income>0** |
| **Interaction** | 2.88e-05 | -0.166* | -0.00718 | 0.00581 | 0.404 | 0.0308 | -0.00178 | 0.627** | -0.0106 |
|  | (0.00316) | (0.0870) | (0.0130) | (0.0145) | (0.340) | (0.0525) | (0.0118) | (0.280) | (0.0382) |
| Exposure Severity | 0.00221 | 0.000897 | 0.991*** | -0.469 | -0.964 | 0.742 | 0.150 | -0.979** | 1.850** |
|  | (0.127) | (0.151) | (0.289) | (0.581) | (0.591) | (1.157) | (0.471) | (0.486) | (0.847) |
| coronavirus year | -0.0164 | 0.363* | 0.00559 | 0.145*** | -0.901 | 0.00976 | 0.0640 | -1.836*** | 0.00869 |
|  | (0.0107) | (0.214) | (0.0509) | (0.0492) | (0.837) | (0.205) | (0.0399) | (0.688) | (0.150) |
| Observations | 8,779 | 977 | 731 | 8,727 | 971 | 725 | 8,726 | 971 | 727 |
| R-squared | 0.899 | 0.897 | 0.916 | 0.624 | 0.640 | 0.599 | 0.637 | 0.676 | 0.655 |
| Note: All controls are the same as full regressions in Table 2 but without education. Robust standard errors in parentheses; *** p<0.01, ** p<0.05, * p<0.1. | | | | | | | | | |

| **Table 10: The Analyses of Hubei Province.** | | | | | | |
| --- | --- | --- | --- | --- | --- | --- |
| **Dependent Variable** | **Life Satisfaction** | **Chronic**  **Disease** | **Sick** | **Sleeping**  **Difficulties** | **Feel Hard to do**  **Everything** | **Loneliness** |
| **Sample** | **Ages of 60 and above** | | | | | |
| coronavirus year | 0.0858 | 0.0773 | -0.0657 | 0.210 | 0.343** | 0.299*** |
|  | (0.117) | (0.0778) | (0.0963) | (0.134) | (0.157) | (0.106) |
| Constant | 5.365*** | 1.466** | 1.338* | 0.794 | 5.524*** | 1.000 |
|  | (0.864) | (0.575) | (0.712) | (0.988) | (1.163) | (0.787) |
|  |  |  |  |  |  |  |
| Observations | 110 | 110 | 110 | 110 | 110 | 110 |
| R-squared | 0.782 | 0.736 | 0.605 | 0.810 | 0.755 | 0.705 |
|  | (1) | (2) | (3) | (4) | (5) | (6) |
| **Dependent Variable** | **Life Satisfaction** | **Chronic**  **Disease** | **Sick** | **Sleeping**  **Difficulties** | **Feel Hard to do**  **Everything** | **Loneliness** |
| **Sample** | **All Sample Surveyed** | | | | | |
| coronavirus year | 0.0213 | 0.0390 | -0.0295 | 0.0827 | 0.186*** | 0.0546 |
|  | (0.0624) | (0.0303) | (0.0392) | (0.0626) | (0.0675) | (0.0475) |
| Constant | 4.021*** | -0.0234 | 0.213 | 0.704 | 0.592 | 3.620*** |
|  | (1.059) | (0.514) | (0.666) | (1.063) | (1.145) | (0.806) |
|  |  |  |  |  |  |  |
| Observations | 484 | 484 | 484 | 484 | 484 | 484 |
| R-squared | 0.752 | 0.693 | 0.653 | 0.759 | 0.664 | 0.735 |

Note: All controls are the same as full regressions in Table 2 and Table 5 and only 2018 and 2020 waves are considered for accurately catching the changes caused by the pandemic. Robust standard errors in parentheses; *** p<0.01, ** p<0.05, * p<0.1.
